# Predictors of Racial Socialization among Black Families: Neighborhood Factors, Racial Discrimination, and Depressive Symptoms

**DOI:** 10.1007/s10826-026-03284-3

**Published:** 2026-04-10

**Authors:** Latisha M. Swygert, Justin A. Lavner, Steven R. H. Beach

**Affiliations:** 1 Department of Psychology, University of Georgia, Athens, GA USA; 2 Department of Psychology, Center for Family Research, University of Georgia, Athens, GA USA

**Keywords:** Racial socialization, Black families, neighborhoods, racial discrimination, depressive symptoms

## Abstract

Racial socialization is a common parenting strategy among Black American caregivers in which they transmit messages about Black American heritage, preparation for discrimination, self- esteem, and connectedness with other racial-ethnic groups. Racial socialization messages foster the development of an understanding of race in Black youth. Racial socialization has been linked to positive outcomes among Black youth, including higher self-esteem and improved mental health outcomes. However, less is known about factors that predict Black caregivers’ use of different racial socialization strategies. To better understand variability in racial socialization messages among Black families, this study examined neighborhood disorder and social cohesion, parents’ and their children’s experiences of racial discrimination, and parents’ depressive symptoms as predictors of caregivers’ transmission of messages of cultural socialization, racial barriers, and preparation for bias using data from a sample of Black families living in the rural Southern U.S. Results revealed several direct and indirect effects and suggest that it is important to further consider linkages between parents’ experience of contextual stressors, their mental health, and their racial socialization practice.

## Highlights

We examined linkages between neighborhood factors, experiences of racial discrimination, depressive symptoms, and racial socialization among Black families.

For Black fathers, contextual stressors might indirectly impact cultural socialization messages through affecting psychological functioning.

Interventions that support Black families may bolster the resilience of Black parents and youth.

Racial discrimination—the unfair and prejudicial treatment of individuals based on racial or ethnic characteristics—is a pervasive stressor for Black Americans across the lifespan. Indeed, approximately 70% of Black adults report daily experiences of racial discrimination (Landrine & Klonoff, 1996; Lee et al., 2019), and approximately 40% of those with these experiences indicate they are prominent stressors ( Dijulio et al., 2015). Additionally, 90% of Black youth report experiencing up to 5 discriminatory experiences daily (English et al., 2020; Pachter et al., 2010). The consequences of experiences of racial discrimination on Black Americans’ mental health are well-documented and include hopelessness, anger, and depressive symptoms (Benner et al., 2018; Carter et al., 2017).

In light of continued racial discrimination and marginalization, many Black caregivers employ parenting strategies such as racial socialization to transmit messages to their children about the significance of race (Hughes, 2003; Hughes et al., 2016; Lesane-Brown, 2006). This important parenting strategy is a mechanism through which youth develop an understanding of race. Several types of racial socialization messages have been identified, including cultural socialization/racial pride, racial barriers, preparation for bias, self-development/self-worth, and egalitarian messages (Hughes et al., 2006; Jones & Neblett, 2017). Cultural socialization, racial barrier, and preparation for bias messages provide teachings about heritage and racial identity and foster awareness of racial discrimination, while self-development and egalitarian messages focus on fostering confidence in individual traits and emphasizing the connectedness that can exist between racial/ethnic groups. Racial socialization messages have been associated with a number of positive outcomes among Black youth, including lower levels of internalizing and externalizing symptoms, greater academic success and efficacy, and higher self-esteem (Umana-Taylor & Hill, 2020).

Given the prevalence and benefits of racial socialization messages for Black youth, understanding factors that predict families’ use of different racial socialization strategies is important to inform theory and practice. The current study seeks to advance knowledge of factors that predict different types of racial socialization messages among Black families living in the rural Southern United States. Specifically, we focus on associations between three types of racial socialization messages—cultural socialization, racial barriers, and preparation for bias—and neighborhood disorder and social cohesion, parents’ and their children’s experiences of racial discrimination, and parents’ depressive symptoms.

## Predictors of Racial Socialization

### Neighborhood Factors

Studies examining the impact of neighborhood factors on parenting within Black families have drawn on a number of theoretical perspectives. Ecological systems theory (Bronfenbrenner, 1986), for example, offers a framework for examining the developing child and their family that posits that family processes such as parental socialization are influenced and shaped directly and indirectly by the systems in which families are embedded. As such, caregivers’ parenting practices may be impacted by factors such as neighborhood disorder. Garcia Coll and colleagues (1996) expanded Bronfenbrenner’s theory in the integrative model of development for racial ethnic minority children to include the variables of race, ethnicity, culture, and social class to appropriately capture family processes within marginalized families. This theory suggests that marginalized families experience systemic barriers based on social position, such that a lower social position results in increased exposure to systems of oppression manifested as factors such as neighborhood disorder or access to resources. Further, Shaw and McKay’s (1942) social disorganization theory posits that neighborhood risk factors such as neighborhood problems and disorder can prevent the development of positive relationships and comradery among neighbors, creating an environment that may also impact parenting practices. Within these perspectives, Black Americans’ parenting practices such as racial socialization may be shaped by the intersection of race and ethnicity and the environment(s) in which the family resides.

Empirical research examining associations between neighborhood characteristics and racial socialization practices among Black families has yielded inconsistent patterns. Caughy et al. (2006) examined how racial socialization practices differ by neighborhood characteristics for Black families living in an urban setting. Findings revealed a positive association between negative neighborhood climates (e.g., physical and/or social disorder) and messages of cultural mistrust and bias preparation, such that greater neighborhood disorder was associated with caregivers placing greater emphasis on issues associated with racism. Similarly, Witherspoon and colleagues (2022) found that Black parents living in urban neighborhoods with lower levels of comradery among residents and higher levels of neighborhood disorder reported more cultural socialization and bias preparation messages. These findings suggest that Black parents may engage in more racial socialization practices to protect their children from risky living environments. In contrast, findings from Witherspoon et al. (2019) indicated that Black families living in “integral” urban neighborhood types characterized by high levels of social cohesion (e.g., trust and connections between neighbors) and low levels of neighborhood problems reported greater transmission of messages of egalitarian beliefs and bias preparation relative to those who live in “anomic” neighborhoods (i.e., low levels of cohesion and problems) and neighborhoods characterized by high levels of neighborhood problems and social processes. Of note, integral neighborhoods were also more likely to be structurally disadvantaged, reflected in higher levels of poverty, suggesting that indicators of structural poverty may contribute to an increase in messages emphasizing preparation for bias. When considered together, these findings suggest that Black caregivers emphasize messages of bias preparation across neighborhood contexts, such that Black youth are prepared for future experiences of discrimination within neighborhoods of high and low levels of cohesion and disorder. However, few studies have used rural samples, leaving open questions about how neighborhood factors such as neighborhood disorder and social cohesion are associated with racial socialization practices in these settings.

### Experiences of Racial Discrimination

Studies examining the impact of perceived racial discrimination experiences on racial socialization practices have also drawn on Bronfenbrenner’s (1986) ecological systems theory, arguing that parents’ and children’s experiences of racial discrimination may influence parents’ transmission of racial socialization messages to their children. Garcia Coll and colleagues (1996) go on to argue that racial and ethnic minority parents engage in an “adaptive culture” of parenting practices that are shaped by experiences such as racial discrimination.

Previous research has shown linkages between children’s experiences of racial discrimination and parents’ racial socialization behaviors. Black parents’ perception of unfair treatment of their children by adults significantly predicted increased messages of bias preparation, but parents’ reports of children’s unfair treatment by peers did not (Hughes & Johnson, 2001). Additionally, both parent- and child- reports of children’s unfair treatment by adults predicted messages promoting mistrust but not the transmission of cultural socialization. Black parents may place more emphasis on communicating with their children about the potential for racial discrimination from adults as opposed to their peers because of the authoritative role that most adults have in children’s lives, as well as the potential for more significant consequences. Conversely, in an examination of parent profiles of racial socialization, White-Johnson et al. (2010) found no differences in the amount of racial socialization messages transmitted in response to child experiences of racial discrimination.

Previous findings also highlight linkages between parents’ own experiences of racial discrimination and racial socialization behaviors. Black parents experiencing more racial discrimination have reported more frequent use of preparation for bias messages compared to parents experiencing less discrimination (Hughes, 2003). Cooper et al. (2015) reported similar findings, such that when parents’ reports of experiences of discrimination were low, transmission of all racial socialization messages was also low (and conversely, when parents reported greater discrimination, they reported transmitting more racial socialization messages). Another study showed that Black parents’ reports of personal experiences of discrimination significantly predicted an increased use of messages of cultural socialization, preparation for bias, and racial barriers (McNeil Smith et al., 2016).

### Parental Depression

To our knowledge, there have been no studies examining linkages between depressive symptoms and racial socialization practices among Black parents. However, the link between depression and other parenting practices has been well-established, such that parenting behavior among parents with higher levels of depression/depressive symptoms differs from that of parents with lower levels of depression/depressive symptoms (Trussell et al., 2018). Common symptoms of depression (e.g., difficulty concentrating, feelings of sadness) may make it more difficult for caregivers to engage with their children. Indeed, higher maternal depressive symptoms have been associated with inconsistent and negative parenting practices (Errazuriz Arellano et al., 2012). Maternal depressive symptoms such as withdrawal and irritability may impact how positively engaged a mother is able to be with her child (Feng et al., 2007; Guyon-Harris et al., 2016; Tully et al., 2017). Studies have shown that mothers with depressive symptoms have reported less involvement and more negative interactions with their children (Feldman et al., 2009; Foster et al., 2008). Further, mothers having difficulty engaging in positive interactions with their children due to their depressive symptoms are more likely to become disengaged, be more irritable, ignore their children, and employ more hostile or negative parenting behaviors such as yelling or using harsh and critical statements (Conners-Burrow et al., 2014; Foster et al., 2008; Milgrom et al., 2014). Similar patterns have been found in fathers, such that higher levels of paternal depressive symptoms have been associated with more negative parenting practices such as authoritarian parenting and spanking (Cameron et al., 2016; Wilson & Durbin, 2010). Among Black fathers specifically, fathers reporting more depressive symptoms exhibited less contact, less closeness, low monitoring, and increased conflict with their children (Davis et al., 2009).

Given the existing literature showing consistent associations between depressive symptoms and other parenting behaviors, there is reason to expect that depressive symptoms may be associated with racial socialization behaviors as well. For example, given that depressive symptoms may create challenges for positive parenting and limit parents’ ability to focus on their children and carry out goal-directed activities (e.g., Dix et al., 2009), Black caregivers experiencing high levels of depressive symptoms may be less able than those with low levels of depressive symptoms to engage in positive parenting behaviors and goal-directed activities seen in cultural socialization messages, such as engaging children in cultural events and activities and conveying messages of pride in their racial group. Additionally, Black caregivers with high levels of depressive symptoms may rely more on the use of negatively toned racial socialization messages when parenting their children, such as preparing them for bias and/or racial barriers.

## Considering Indirect Effects

In addition to possible bivariate associations between Black parents’ depressive symptoms and racial socialization, parents’ depressive symptoms might also serve to link contextual stressors like neighborhood disadvantage and racial discrimination to racial socialization. That is, stressors such as neighborhood disorder or experiences of racial discrimination may predict Black parents’ depressive symptoms and, in turn, predict their racial socialization practices. These types of linkages would be consistent with the general tenets of the Family Stress Model (e.g., McLoyd, 1990), in which contextual stressors affect parenting through their effects on parents’ psychological functioning. Findings from Hill and Herman-Stahl (2002) lend tentative support to this idea: maternal depressive symptoms mediated the relationship between neighborhood characteristics and negative parenting styles, suggesting that increased psychological distress from disorganized and unsafe neighborhoods may indirectly impact mothers’ ability to employ positive parenting styles. As such, Black caregivers experiencing high levels of depressive symptoms as a result of greater perceived neighborhood disorder and/or high levels of racial discrimination may be more likely to employ racial socialization messages that promote safety behaviors, such as preparation for bias. Further examination of these types of indirect effects is needed.

## The Present Study

The present study aimed to advance understanding of several factors that may predict the use of three types of racial socialization messages— cultural socialization, racial barriers, and preparation for bias—among Black families living in the rural Southern United States (US). In so doing, we aimed to both replicate and extend previous work in this area. We examined the following research questions (RQs):

(RQ1) How are neighborhood disorder and social cohesion associated with families’ use of different racial socialization messages?

(RQ2) How are parents’ and their child’s experiences of racial discrimination associated with families’ use of different racial socialization messages?

(RQ3) How are parents’ depressive symptoms associated with families’ use of different racial socialization messages?

(RQ4) Are there significant indirect effects from neighborhood disorder and social cohesion and/or parents’ experiences of racial discrimination to families’ use of different racial socialization messages through parents’ depressive symptoms?

## Method

### Participants and Procedure

Families were recruited for the Protecting Strong African American Families.

(ProSAAF) project, a clinical trial of a family-centered intervention to enhance couple, coparenting, and parent–child relationships in Black families living in the rural, southern United States (see Barton et al., 2018). The present study uses data from the project’s baseline assessment (i.e., pre-intervention). Couples with an African American child between the ages of 9 and 14 years took part in the study. The study received approval from the Institutional Review Board at the University of Georgia (study title: Protecting Strong African American Families; institutional review board approval number: 2012104112). Subject enrollment began in 2013 and continued into 2014. All participants lived in small towns and communities in the Southeastern US, where poverty rates are among the highest in the nation and unemployment rates are above the national average (DeNavas-Walt & Proctor, 2014). To be eligible, couples had to be in a relationship for 2 years or more, living together, and coparenting an African American child in the targeted age range for at least 1 year. Given the aims of the broader study, families had to be willing to spend 6 weeks engaged in a family-centered prevention program and not be planning to move out of the study area during that period.

Families were recruited by mail and phone via advertisements distributed in their communities as well as through lists that local schools provided. Schools in 16 counties provided information on youths in grades 4 through 6. A total of 1897 families were screened for eligibility. Of these families, 1145 were ineligible (e.g., household was headed by a single parent, the child was not in the targeted age range, the child was not African American, the family was enrolled in another program, the child had a sibling/stepsibling in the same grade). Of the 752 eligible families, 347 did not respond to the solicitation and 59 were unable to schedule an assessment. The remaining 346 families completed baseline (i.e., pre-intervention) assessments.

Of the baseline sample, 63% of couples were married, with a mean length of marriage of 9.8 years (*SD* = 7.48; range < 1 year to 56 years). Unmarried couples had been living together for an average of 6.7 years (*SD* = 5.42; range < 1 year to 24 years). Adults’ mean ages were 39.9 years (*SD* = 9.6; range 21 to 83 years) for men and 36.6 years (*SD* = 7.5; range 23 to 73 years) for women. Children’s mean age was 10.9 (*SD* = 0.9; range 9 to 14 years). The majority of families (51%) had incomes below 100% of the federal poverty level and an additional 17% had incomes between 100% and 150% of that level. The majority of both men (74% [65% full-time]) and women (61% [45% full-time]) were employed. Median monthly income was $1,375 (*SD* = $1,375; range $1 to $7,500) for men and $1,220 (*SD* = $1,440; range $1 to $10,000) for women. Median education levels were high school or GED (ranging from less than grade 9 to a doctorate or professional degree) for men and some college or trade school (ranging from less than grade 9 to a master’s degree) for women. Nearly all couples were mixed-sex (*n* = 344 [99.4%]); two families were headed by a female same-sex couple and were excluded from analyses given our focus on effects for mothers and fathers.

Project staff visited couples’ homes, explained the study in greater detail, and obtained informed consent. Each participating family member then completed the baseline assessment using audio computer-assisted self-interview software installed on laptop computers. Participants completed surveys on separate laptops and, if possible, in separate rooms. Participants did not talk to one another or see one another’s responses while completing the surveys. Each adult was compensated with a $50 check and youth received a $20 gift card.

### Measures

#### Parental Racial Socialization

Parental racial socialization was measured using the Racial Socialization Scale (RSS; Hughes & Johnson, 2001). The RSS is a 15-item youth-report measure that assesses the number of times their parents engaged in specified socialization behaviors during the past month. We assessed three racial socialization dimensions: Cultural socialization (5 items; sample item: “How often in the past month have your parents celebrated cultural holidays of your racial group?”), Preparation for bias (6 items; sample item: “How often in the past month have your parents told you that people might keep you from doing well because of your race?”), and Racial barriers (4 items; sample item: “How often in the past month have your parents told you not to trust kids from other racial or ethnic groups?”). Response options ranged from 1 = *never* to 3 = *three to five times*. Items from each dimension were summed to create total subscale scores, which exhibited good internal consistency: Cultural socialization (5 items; α = 0.75), Preparation for bias (6 items; α = 0.80), and Racial barriers (4 items; α = 0.75).

#### Neighborhood Factors

Neighborhood social cohesion was measured using the Neighborhood Social Cohesion Scale (Sampson et al., 1997). This 6-item measure, completed separately by each parent, assessed the level of social support among individuals within the families’ neighborhoods (sample item: in the neighborhood surrounding your house…“People are willing to help each other out”), with responses ranging from 1 = *not at all true* to 3 = *very true*. Responses were summed, with higher scores indicating greater social cohesion. Internal consistency was acceptable (mothers: α = 0.78, fathers: α = 0.68).

Neighborhood disorder was assessed using 6 items from the neighborhood disorder subscale of the Community Deviance Scale (Simons et al., 1995). Each parent reported on how much of a problem disorder was in their neighborhoods (sample item: “How much of a problem is “people selling or using drugs” in your neighborhood”), with responses ranging from 0 = *a big problem* to 2 = *not a problem*. Scores were reverse coded and summed, with higher scores indicating more problems. Internal consistency was high (mothers: α = 0.90, fathers: α = 0.92).

#### Experiences of Racial Discrimination

Participants’ experiences of racial discrimination were assessed using 9 items from the daily life experiences subscale of the Racism and Life Experiences Scale (Harrell et al., 1997). Participants were asked to report the frequency with which they experienced several racial stressors over the last 6 months (sample items: “Have you been treated rudely or disrespectfully because of your race?”; “Have you been called a name or harassed because of your race?”) using a four-point Likert scale (1 = *never*, 2 = *once or twice*, 3 = *a few times*, 4 = *frequently*). Responses were summed separately for each parent and the child, with higher scores indicating more racial discrimination. Internal consistency was high (mothers: α = 0.90; fathers: α = 0.92; youth: α = 0.85).

#### Parental Depressive Symptoms

Parents reported on their depressive symptoms using the Center for Epidemiologic Studies Depression Scale (CES-D; Radloff, 1997). This 20-item measure assessed how often participants had experienced depressive symptoms over the past week (sample item: How often in the last week “did you feel depressed”), with responses ranging from 0 = *rarely or none of the time (0–1 day)* to 3 = *most or all of the time (6–7 days)*. Scores were summed separately for mothers and fathers, with higher scores reflecting more depressive symptoms. Internal consistency was good (mothers: α = 0.85; fathers: α = 0.75).

### Data Analytic Plan

To test RQ1-3 examining associations between various predictor variables and the three racial socialization messages (i.e., racial barriers, preparation for bias, cultural socialization) we conducted a series of multivariate regression analyses using SPSS. In each model, the predictor was entered as the independent variable and the three racial socialization messages were entered as dependent variables. To test RQ4 examining indirect effects, we used the PROCESS macro (Version 4; Model 4) in SPSS (Hayes, 2022). Indirect effect models were tested using 5,000 bootstrap estimates to generate 95% confidence intervals (CIs).

## Results

### Preliminary Analyses

Descriptive statistics for and correlations among study variables are shown in Table 1. Amongst the three racial socialization messages, cultural socialization was significantly positively associated with racial barriers (*r*(342) = 0.25, *p* < .001) and preparation for bias (*r*(341) = 0.46, *p* < .001), such that youth who reported receiving more cultural socialization messages from their parents also reported receiving more racial barriers and preparation for bias messages. Racial barriers messages were also significantly positively associated with preparation for bias messages (*r*(341) = 0.49, *p* < .001), indicating that youth who reported receiving more messages regarding racial barriers from their parents also reported receiving more messages of preparation for bias. Paired sample *t*-tests comparing mothers’ and fathers’ scores on neighborhood social cohesion, neighborhood disorder, racial discrimination, and depressive symptoms indicated that parents differed in their reports of neighborhood disorder (*t*(341) = -3.43, *p* < .001) and racial discrimination (*t*(343) = -6.29, *p* < .001)—fathers reported more neighborhood disorder and racial discrimination than did mothers—but not in their reports of neighborhood social cohesion or depressive symptoms (both *p* > .05).

**Table 1:** Descriptive statistics for and correlations among study variables

Variable	1	2	3	4	5	6	7	8	9	10	11	12
1. Racial barriers (Y)	—											
2. Preparation for bias (Y)	0.49***	—										
3. Cultural socialization (Y)	0.25***	0.46***	—									
4. Social cohesion (Mo)	− 0.01	0.06	0.04	—								
5. Neighborhood disorder (Mo)	0.01	− 0.01	0.04	− 0.19***	—							
6. Social cohesion (Fa)	0.04	0.05	− 0.03	0.30***	− 0.11*	—						
7. Neighborhood disorder (Fa)	0.03	− 0.08	− 0.14*	− 0.17**	0.16**	− 0.10	—					
8. Racial discrimination (Mo)	− 0.00	0.04	0.01	− 0.12*	0.13*	− 0.03	− 0.09	—				
9. Racial discrimination (Fa)	− 0.02	0.00	− 0.05	− 0.01	− 0.00	− 0.05	0.10	0.10	—			
10. Racial discrimination (Y)	0.32***	0.35***	0.15**	0.07	0.08	− 0.05	− 0.02	0.05	0.04	—		
11. Depressive symptoms (Mo)	0.02	− 0.06	− 0.05	− 0.17**	0.12*	− 0.04	0.02	0.11*	0.02	− 0.01	—	
12. Depressive symptoms (Fa)	− 0.07	− 0.07	− 0.14**	− 0.03	0.06	− 0.18**	0.20***	0.01	0.22***	− 0.03	0.12*	—
*N*	344	344	344	343	343	343	342	344	344	344	344	344
*M*	5.08	8.43	8.87	14.01	3.01	14.20	3.93	14.88	17.59	13.41	12.01	10.97
*SD*	1.75	2.74	2.87	2.76	3.63	2.36	4.16	5.30	6.54	4.93	8.50	6.84

Mo = reported by the mother; Fa = reported by the father; Y = reported by the youth

*p < .05. **p < .01. ***p < .001

### RQ1: Neighborhood Factors and Racial Socialization Messages

Fathers’ reports of neighborhood disorder were significantly negatively associated with youth-reported cultural socialization messages (B = − 0.093, SE = 0.04, *p* = .012), such that when fathers reported more neighborhood disorder, their child reported receiving fewer cultural socialization messages from their parents (Table 2). No other significant associations with the three racial socialization messages were found for fathers’ reports of neighborhood disorder or social cohesion, or for mothers’ reports of neighborhood disorder or social cohesion (all *p* > .05).

**Table 2:** Multivariable regression models predicting racial socialization

Predictor	Racial barriers			Preparation for bias			Cultural socialization	
	B(SE)	*p*-value		B(SE)	*p*-value		B(SE)	*p*-value
Social cohesion (Mo)	− 0.007 (0.03)	0.839		0.057 (0.05)	0.293		0.039 (0.06)	0.487
Neighborhood disorder (Mo)	0.005 (0.03)	0.860		− 0.009 (0.04)	0.833		0.030 (0.04)	0.476
Social cohesion (Fa)	0.027 (0.04)	0.500		0.058 (0.06)	0.355		− 0.037 (0.07)	0.568
Neighborhood disorder (Fa)	0.014 (0.02)	0.533		− 0.051 (0.04)	0.154		**− 0.093 (0.04)**	**0.012**
Racial discrimination (Mo)	− 0.001 (0.02)	0.964		0.019 (0.03)	0.500		0.003 (0.03)	0.928
Racial discrimination (Fa)	− 0.005 (0.01)	0.713		0.001 (0.02)	0.982		− 0.021 (0.02)	0.382
Racial discrimination (Y)	**0.113 (0.02)**	**< 0.001**		**0.194 (0.03)**	**< 0.001**		**0.084 (0.03)**	**0.007**
Depression symptoms (Mo)	0.004 (0.01)	0.726		− 0.018 (0.02)	0.293		− 0.017 (0.02)	0.358
Depressive symptoms (Fa)	− 0.017 (0.01)	0.212		− 0.028 (0.02)	0.194		**− 0.059 (0.02)**	**0.009**

Each predictor was analyzed in a separate multivariable regression model that included all three racial socialization variables as outcomes. Significant results are bolded for emphasis. Mo = reported by the mother; Fa = reported by the father; Y = reported by the youth

### RQ2: Experiences of Racial Discrimination and Racial Socialization Messages

There were significant positive associations between children’s experiences of racial discrimination and racial barriers messages (B = 0.113, SE = 0.02, *p* < .001), preparation for bias messages (B = 0.194, SE = 0.03, *p* < .001), and cultural socialization messages (B = 0.084, SE = 0.03, *p* = .007), such that youth experiencing more racial discrimination reported that their parents engaged in higher levels of all three racial socialization dimensions (Table 2). Mothers’ and fathers’ experiences of racial discrimination were not significantly associated with youth-reported racial socialization messages (all *p* > .05).

### RQ3: Parents’ Depressive Symptoms and Racial Socialization Messages

Fathers’ depressive symptoms were significantly negatively associated with cultural socialization messages (B = − 0.059, SE = 0.02, *p* = .009), such that when fathers reported more depressive symptoms, their child reported receiving fewer cultural socialization messages from their parents (Table 2). There were no significant associations between fathers’ depressive symptoms and the other dimensions of racial socialization, or between mothers’ depressive symptoms and the three different racial socialization messages (all *p* > .05).

### RQ4: Indirect Effects

Given significant associations between fathers’ depressive symptoms and cultural socialization messages, we explored the possibility of this pathway resulting in indirect effects. To do so, we first examined whether fathers’ ratings of neighborhood factors and their racial discrimination experiences were significantly associated with their depressive symptoms. These results indicated that fathers’ reports of neighborhood social cohesion were significantly negatively associated with their depressive symptoms (*r*(341) = − 0.18, *p* = .001), and their reports of neighborhood disorder (*r*(340) = 0.20, *p* < .001) and their experiences of racial discrimination (*r*(342) = 0.22, *p* < .001) were significantly positively associated with their depressive symptoms.

In light of these significant associations, we proceeded by testing three indirect effects: (1) fathers’ reports of neighborhood social cohesion to fathers’ depressive symptoms to cultural socialization messages, (2) fathers’ reports of neighborhood disorder to fathers’ depressive symptoms to cultural socialization messages, and (3) fathers’ experiences of racial discrimination to fathers’ depressive symptoms to cultural socialization messages. Results of the indirect effect models are shown in Table 3. There was a significant positive indirect effect from fathers’ reports of neighborhood social cohesion to cultural socialization through fathers’ depressive symptoms (IE = 0.0319, 95% CI [0.0076, 0.0631]): higher levels of social cohesion were associated with lower levels of depressive symptoms, which were associated with higher levels of cultural socialization. Additionally, there was a significant negative indirect effect from fathers’ reports of neighborhood disorder to cultural socialization through fathers’ depressive symptoms (IE = − 0.0186, 95% CI [-0.0361, − 0.0045]): higher levels of neighborhood disorder were associated with higher levels of depressive symptoms, which were associated with lower levels of cultural socialization. Similarly, there was a significant negative indirect effect from fathers’ experiences of racial discrimination to cultural socialization through fathers’ depressive symptoms (IE = − 0.0130, 95% CI [-0.0268, − 0.0026]): higher levels of racial discrimination were associated with higher levels of depressive symptoms, which were associated with lower levels of cultural socialization. A significant direct effect was also found for fathers’ reports of neighborhood disorder on cultural socialization (b = − 0.07, *p* < .001), such that higher levels of neighborhood disorder were associated with lower levels of cultural socialization.

**Table 3:** Indirect effects of fathers’ reports of social cohesion, neighborhood disorder, and racial discrimination on cultural socialization through depressive symptoms

		Indirect effect through depressive symptoms (a x b)			Direct effect (c’)		
Predictor	*N*	Coefficient	Boot SE	95% CI	Coefficient	se	*p*
Social cohesion	343	0.0319	0.0141	[0.0076, 0.0631]	− 0.0693	0.0659	0.2937
Neighborhood disorder	342	− 0.0186	0.0082	[-0.0361, − 0.0045]	− 0.0743	0.0372	0.0462
Racial discrimination	344	− 0.0130	0.0062	[-0.0268, − 0.0026]	− 0.0077	0.0240	0.7483

Unstandardized coefficients shown. CI = confidence interval

## Discussion

The present study sought to build on previous work examining factors associated with the transmission of racial socialization messages among Black American families by examining linkages between neighborhood characteristics, parents’ and children’s experiences of racial discrimination, parents’ depressive symptoms, and racial socialization messages. Notably, our sample included data from rural Black families, building on prior work which has focused primarily on urban samples.

Regarding neighborhood factors and parents’ transmission of racial socialization messages, there was only one significant bivariate association: a negative association between fathers’ reports of neighborhood disorder and youth-reported cultural socialization messages. This indicates that in families where fathers reported higher levels of neighborhood disorder, their child reported fewer cultural socialization messages. This pattern is consistent with conceptual models that point to the importance of considering the systems in which Black families reside. The integrative model of development for racial ethnic minority children (Garcia Coll et al., 1996) theorizes that marginalized families experience increased exposure to systemic manifestations of oppression through factors such as risky neighborhood environments. Further, social disorganization theory (Shaw & McKay, 1942) suggests that these environments may influence parenting practices through shared parenting by neighboring parents who assist in monitoring neighborhood children’s behaviors in risky environments. Parents in risky neighborhood environments may rely more heavily on community formed in these environments for support. Thus, it is possible that Black parents may rely on the transmission of racial socialization messages by other parents and adults in their neighborhoods resulting in a lower transmission of racial socialization messages from parents to their own children.

Notably, no other significant associations were found between mothers’ or fathers’ reports of neighborhood disorder or social cohesion and the three racial socialization messages. Previous research examining the relationship between neighborhood characteristics and racial socialization practices has been mixed, though most studies have found more consistent evidence of linkages between neighborhood characteristics and racial socialization practices than we found here. Some studies have found that Black parents reporting high neighborhood disorder report greater transmission of all racial socialization messages, whereas other studies have found evidence for increased emphasis on messages of mistrust and bias preparation in the context of high neighborhood disorder. Our study, however, only revealed significant effects for fewer cultural socialization messages when Black fathers reported higher levels of neighborhood disorder, as opposed to an increase in transmission of all racial socialization messages or more negatively toned messages (i.e., cultural mistrust, preparation for bias). It is possible that differences in setting contribute to different patterns across studies: to our knowledge, few studies have examined the linkages between neighborhood contexts and the transmission of racial socialization messages in rural settings as we did here. These findings suggest that it may be important to consider differences and similarities between rural and urban communities when examining associations between neighborhood risk factors and Black families’ parenting practices; parents’ transmission of racial socialization messages may depend on the norms and values shared among residents in a particular setting (urban or rural areas).

Regarding racial discrimination, there were significant positive associations between children’s experiences of discrimination and their reports of their parents’ transmission of preparation for bias, racial barriers, and cultural socialization messages. Black parents appear to emphasize a variety of teachings about race to their children in response to more reported experiences of racial discrimination, bolstering their children’s Black identity (cultural socialization) while simultaneously preparing them for inevitable unfair treatment on the basis of race (bias preparation and racial barriers). These results are consistent with previous work indicating associations between Black parents’ emphasis of all racial socialization messages and their children’s reported experiences of discrimination (White-Johnson et al., 2010) and with conceptual models that theorize that minority parents adapt their parenting practices in response to experiences such as racial discrimination (Garcia Coll et al., 1996).

Surprisingly, there were no significant associations between parents’ own experiences of racial discrimination and racial socialization practices, counter to previous work (Cooper et al., 2015; McNeil Smith et al., 2016). It is possible that Black parents are more likely to increase their transmission of racial socialization messages in response to their children’s experiences of racial discrimination to ensure that their children are prepared for future experiences and are equipped with knowledge to combat negative feelings associated with experiencing racial discrimination. Additionally, there may be differences between children’s reports of their parents’ racial socialization messages (as used in the current study) and parents’ reports of their racial socialization practices, which have been used in other studies (Cooper et al., 2015; Hughes, 2003).

Regarding parents’ depressive symptoms, there was a significant negative association between fathers’ depressive symptoms and cultural socialization messages. No other significant associations were found for fathers’ depressive symptoms and bias preparation or racial barrier messages, or between mothers’ depressive symptoms and the three dimensions of racial socialization messages. Though there have been no studies to our knowledge examining linkages between depressive symptoms and racial socialization practices among Black parents, these findings are consistent with studies examining parenting behavior and depression. Maternal depressive symptoms have been associated with less involvement with their children (Feldman et al., 2009), and paternal depressive symptoms have been associated with less engagement and increased conflict with their children (Davis et al., 2009). As such, it is possible that Black fathers with higher depressive symptoms experience difficulty engaging their children in goal-directed activities associated with cultural socialization (e.g., attending cultural events). However, it is important to note that these associations were not significant among mothers. It is possible that mothers receive sources of social support that fathers do not (e.g., from other mothers, women in their families, etc.) when experiencing difficulties that may otherwise interfere with their parenting (Bost et al., 2002; Hughes et al., 2020).

In addition to these direct (bivariate) effects, our findings revealed significant indirect effects as well. Specifically, several indirect effects through fathers’ depressive symptoms were found: a significant positive indirect effect from fathers’ reports of neighborhood social cohesion to cultural socialization, a significant negative indirect effect from fathers’ reports of neighborhood disorder to cultural socialization, and a significant negative indirect effect from fathers’ experiences of racial discrimination to cultural socialization. These results indicate that higher levels of social cohesion, lower levels of neighborhood disorder, and lower levels of racial discrimination experiences were associated with lower levels of fathers’ depressive symptoms, which were associated with higher levels of cultural socialization. Taken together, our findings suggest that contextual stressors such as neighborhood risk factors and racial discrimination might indirectly predict Black parenting practices through depressive symptoms. This is consistent with theories that posit that parenting practices may be impacted by parents’ psychological wellbeing (McLoyd, 1990).

Before considering the implications of these findings, it is important to note that this study is not without limitations. First, the data used were cross-sectional, so the direction of effects cannot be determined. Future studies should examine these patterns using longitudinal study designs. Second, the focal youths were pre-adolescents and so it is unclear how the results might apply to other developmental periods among Black youth. Future studies including both younger and older children would be valuable. Third, as noted previously, the measure of racial socialization behaviors was completed by youth about their parents, and may differ from parents’ reports. Future studies should include both types of measures. Fourth, although our design was novel in that it examined these associations within a rural community, participants in the sample all resided within the same region. It is possible that the patterning of the relationship between contextual stressors, parents’ depressive symptoms, and racial socialization in Black parents would differ across rural communities.

Notwithstanding these limitations, these findings have important implications. Structural racism continues to negatively impact Black parents and their children through increasing neighborhood disadvantage and exposure to interpersonal racial discrimination. These findings indicate the importance of considering indirect linkages between these contextual stressors and racial socialization parenting practices among Black parents. For Black fathers specifically, exposures to contextual stressors such as neighborhood risk factors and racial discrimination may interfere with their ability to engage in positive cultural socialization practices through increasing depressive symptoms. Identifying supports for Black fathers may be helpful in lessening the impacts of racial discrimination and neighborhood risk factors. Specifically, clinicians should be aware of the impact of structural disadvantages on Black parents and provide supports (e.g., stress management skills, coping skills, assistance with access to parental resources) that address the relations between contextual stressors, depressive symptoms, and parenting practices. Doing so would bolster Black parents’ resilience and their ability to provide positive aspects of racial socialization known to be important for Black youth. Intervention efforts might also target improving the parent-child relationship to lessen the impact of contextual stressors. More broadly, there is an urgent need to dismantle structural disadvantages for Black Americans. For example, systemic interventions such as increasing investment in disadvantaged neighborhoods have been shown to benefit Black Americans’ health (e.g., Dubowitz et al., 2021) and may have downstream benefits for family functioning as well through increasing families’ ability to engage in positive activities together (e.g., Lavner, 2025). Reducing and ultimately eliminating systemic barriers and discriminatory experiences would enhance the health and well-being of Black families.
